# Chickenpox and Risk of Stroke: A Self-controlled Case Series Analysis

**DOI:** 10.1093/cid/cit659

**Published:** 2013-10-02

**Authors:** Sara L. Thomas, Caroline Minassian, Vijeya Ganesan, Sinéad M. Langan, Liam Smeeth

**Affiliations:** 1 Faculty of Epidemiology and Population Health, London School of Hygiene and Tropical Medicine; 2 Institute of Child Health, University College London, United Kingdom

**Keywords:** chickenpox, stroke, child, adult, risk factors

## Abstract

***Background.*** There is good evidence that respiratory and other infections that cause systemic inflammation can trigger strokes; however, the role of specific infections is unclear. Case reports have highlighted chickenpox as a possible risk factor for arterial ischemic stroke, particularly in children, but rigorous studies are needed to determine and quantify any increased risk.

***Methods.*** We used anonymized electronic health records totaling >100 million person-years of observation from 4 UK primary care databases to identify individuals who had documented clinical chickenpox and a stroke or transient ischemic attack (TIA). Self-controlled case series methods were used to quantify any increased risk of first stroke or TIA in the 0–6 and 7–12 months following chickenpox compared to other observed time periods. We analyzed data within each database, and performed meta-analyses to obtain summary age-adjusted incidence ratios (IRs) separately for adults and children.

***Results.*** Five hundred sixty eligible individuals (including 60 children) were identified who experienced chickenpox and a stroke or TIA during follow-up. Among children, there was a 4-fold increased risk of stroke in the 0–6 months after chickenpox (summary IR = 4.07; 95% confidence interval [CI], 1.96–8.45; *I*^2^ = 0%). Among adults, there was a less marked increased risk with moderate between-database heterogeneity (random-effects summary IR = 2.13; 95% CI, 1.05–4.36; *I*^2^ = 51%). There was no significant increased risk of stroke in the 7–12 months after chickenpox in children or adults, nor was there evidence of increased risk of TIA in either time period.

***Conclusions.*** Our study provides new evidence that children who experience chickenpox are at increased risk of stroke in the subsequent 6 months.

## Abstract (precis)

Children who experience chickenpox are at a 4-fold increased risk of ischemic stroke in the subsequent 6 months. The evidence is less strong for adults, possibly due to mechanistic differences in the role of inflammation in adult stroke risk.

**(See the Editorial Commentary by Gershon on pages 69–71.)**

There is increasing epidemiological evidence that acute infections can trigger arterial ischemic stroke (AIS) [1]. For example, we have shown that acute systemic respiratory and urinary tract infections were each associated with an increased stroke risk in the 3 months after infection [2]. Suggested mechanisms include atherosclerotic plaque rupture and embolism, increased local coagulability, and impairment of endothelial function [1, 3, 4]. Unlike adult strokes, childhood AISs are nonatheromatous; the relationship between infection and pediatric AIS has been less investigated, but a recent US case-control study reported that a medical consultation for minor acute infection was associated with a 4.6-fold increased risk of childhood AIS in the subsequent month [5]. Antecedent infection is also significantly associated with focal intracranial arteriopathy, a common finding in childhood AIS [6]. Thus, as well as the mechanisms postulated in adults, these data suggest that infection may induce focal intracranial arteriopathy, probably inflammatory, in some children with AIS.

Although broad categories of infections have been shown to be associated with increased stroke risk, few studies have investigated the role of specific pathogens. One infectious agent identified as a possible trigger for AIS is varicella zoster virus (VZV), which can replicate in cerebral arterial walls and thus is a plausible cause of cerebral arteriopathy [7]. Cases of childhood AIS or transient ischemic attack (TIA) occurring in the months after chickenpox (primary VZV infection) have been thought to represent a vasculitis, resulting either from direct viral invasion or as a postinfectious phenomenon [8–10]. Cases of AIS following chickenpox have also been reported in adults, and the purported effect may not be restricted to children [11–14].

Because chickenpox is common among children, individual case reports of chickenpox prior to stroke could be explained by chance and do not provide clear evidence of increased risk. Rigorous epidemiological studies are thus needed to quantify any increased risk of AIS following chickenpox. The challenge is that children and adults with and without chickenpox differ in ways that are difficult to capture and control for. In addition, very large prospective data are needed. We therefore used UK electronic health records and the self-controlled case series method to eliminate between-person confounding and test the hypothesis that children and adults who develop chickenpox are at increased risk of AIS in the subsequent 12 months.

## METHODS

### Data Sources

We combined data from 4 large UK general practice databases of anonymized electronic health records, totaling >100 million person-years of observation: (1) the General Practice Research Database (GPRD), one of the world's largest and best-established research databases of electronic primary care data; (2) QResearch, which holds data for >12 million patients; (3) The Health Improvement Network (THIN), with >9 million patients; and (4) IMS Disease Analyzer Mediplus, with >2 million patients. These databases include information on patients' consultations, diagnoses, prescriptions, and outcomes of hospitalizations/specialist referrals, and all have been widely used for epidemiological research.

### Study Design

The within-person self-controlled case series method utilizes data only from cases (individuals with stroke) and allows estimation of the relative incidence of a stroke in defined intervals following acute exposures (chickenpox) compared to other periods in the same individuals [15]. This study design provided 3 major advantages for our study question. First, as comparisons are made entirely within individuals, between-person confounding was addressed. Second, not all individuals with chickenpox attend their general practitioner, but because the method uses only exposed cases (individuals who experienced both chickenpox and stroke), underascertainment of chickenpox was avoided. Third, stroke in childhood is uncommon, but the design is statistically efficient and thus increases study power [15].

### Study Period

Individuals' follow-up started at the later of the date they registered with the practice and either the date from which the data met database-defined quality standards (GPRD and QResearch), practice computerization date (THIN), or 1 January 1990 (IMS Health: a conservative estimate of practice computerization date). Because diagnoses recorded in the first year of follow-up in primary care databases can be past events recorded retrospectively, each individual's study period started 1 year after the start of their follow-up and ended at the earliest of the date they died, left the practice, or the practice's last data collection date (up to January 2011, depending on the database) [16]. Thus, individuals were at least 1 year of age at the study start.

### Exposure and Outcome

Medical records with chickenpox, stroke, and TIA diagnoses were identified using Read code lists (Supplementary Tables 1 and 2). To distinguish strokes from TIAs, we combined all stroke and TIA records into episodes; records occurring within 28 days of one another were considered to be part of an ongoing episode. The episode was then categorized as either a TIA or a stroke using the Read codes within that episode; a TIA episode comprised only TIA records. Episodes with codes indicating a hemorrhagic or venous stroke were also identified for later exclusion. We restricted analyses to the first stroke episode in the study period (the index stroke) to avoid the possibility that a subsequent stroke “episode” was an ongoing (not a new) stroke and to fulfill the assumption of self-controlled case series methods that events should be independent within a person [15].

### Eligibility Criteria

Potentially eligible individuals were those who had a first-ever clinical diagnosis of both chickenpox and stroke (or TIA) during their study period. We excluded individuals who had documented chickenpox or stroke/TIA before the study period, or on the same day as a “new-patient” or “well-patient” health check, which could be a recording of a past event. To restrict the outcome to AIS, we excluded individuals whose first stroke episode was classified as a hemorrhagic or venous stroke, and individuals whose first stroke was documented entirely with nonspecific diagnostic codes (eg, “cerebrovascular accident”) but who also had a risk factor for hemorrhagic stroke anywhere in their medical data—for example, a cerebral arteriovenous malformation or a nontraumatic subarachnoid hemorrhage (as a proxy for an arteriovenous malformation/aneurysm). Finally, meningitis and encephalitis are rare complications of chickenpox, and their initial presentation can mimic that of a stroke. We therefore excluded any individual who had a varicella-specific or nonspecific meningitis or encephalitis diagnostic code in the year after their stroke diagnosis.

General practices that contribute data to QResearch or to IMS are mutually exclusive and do not contribute to the other databases. Some practices contribute data to both GPRD and to THIN and so potentially some patients with chickenpox and stroke could have been duplicated in the GPRD and THIN datasets. We identified GPRD/THIN duplicated cases on the basis of year of birth, sex, geographical region of the practice, and dates of chickenpox and stroke, and excluded these cases from the THIN dataset.

### Analysis

**Figure 1.**
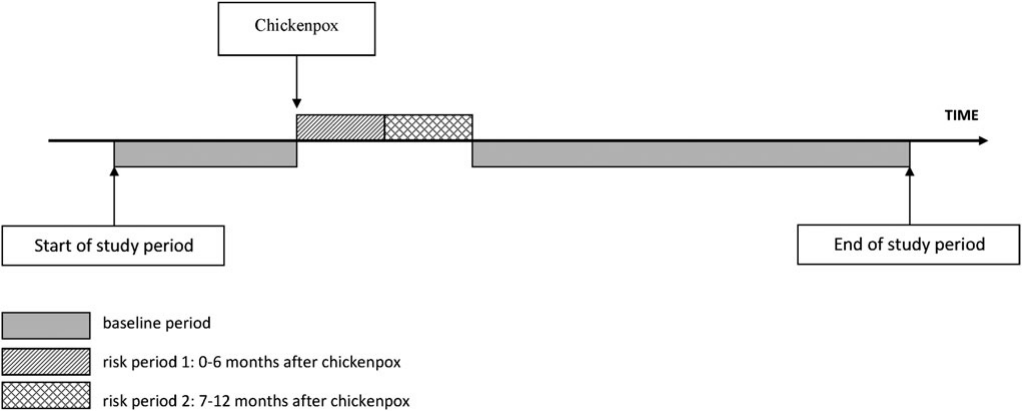
Pictorial representation of self-controlled case-series study design, showing the risk periods and baseline periods for a single patient whose first stroke could occur at any time during the study period.

Self-controlled case series analyses were first carried out separately for each of the 4 datasets. The “exposed” period for each individual started the day after their first chickenpox consultation and extended up to a year, consistent with previous case reports; we subdivided this exposed period into 0–6 months and 7–12 months after chickenpox. All remaining time in an individual's study period was considered “unexposed” baseline (Figure 1). We used conditional Poisson regression to calculate incidence ratios (IRs) for the first-ever stroke or TIA occurring in each of the 2 exposed periods compared to baseline, adjusting for age in 5-year age bands. Age-adjusted analyses were stratified into 2 age groups: Those who experienced both chickenpox and stroke/TIA before 18 years of age contributed to the “child” stratum (with censoring at 18 years), and those experiencing both these events at ≥18 years were included in the “adult” stratum (starting follow-up at 18 years). We examined the effect of chickenpox on any cerebrovascular event (stroke or TIA), and separately on strokes and TIAs.

We investigated heterogeneity between the database-specific IRs using the Cochrane Q statistic and *I*^2^ statistic [17]. When there was no evidence of heterogeneity, we combined the data using fixed-effect meta-analysis to obtain a summary IR; we also combined the data into a single dataset and repeated case series analyses, comparing the IR obtained from the combined dataset with that from the meta-analysis. When evidence of heterogeneity was found, a random-effects meta-analysis was used to obtain a summary IR and the data were not further combined.

Two key assumptions of the self-controlled case series method are that the observation period for each individual is independent of the timing of their clinical event, and that the occurrence of the clinical event does not affect the likelihood of subsequent exposure to the risk factor [15]. These assumptions may not hold for an event such as stroke that increases the mortality rate, because subsequent exposure to chickenpox is impossible after death and the observation period is censored at death and so is event-dependent. To assess whether this had affected our study results, we carried out a sensitivity analysis after excluding individuals whose study period ended within a month of their stroke (possibly indicating death).

All analyses were carried out using Stata software, release 12 (StataCorp, College Station, Texas).

### Ethics Approval

The study received approval from the scientific/ethics advisory group of the 4 databases, and from the ethics committee of the London School of Hygiene and Tropical Medicine.

## RESULTS

We identified from the 4 databases 645 potentially eligible cases with a first-ever recorded stroke or TIA and a first chickenpox consultation during their study period. Of these, 77 were excluded: 26 had their first stroke and/or first chickenpox record on the same day as a new- or well-person check-up, 50 had a hemorrhagic or venous first stroke, 1 had evidence of a subarachnoid hemorrhage, and 1 had a varicella meningitis record 24 days after their first stroke. Eight further individuals were excluded because they had chickenpox outside their age-determined observation period (had stroke or TIA as an adult but chickenpox as a child, or vice versa).

Characteristics of the 560 remaining eligible individuals are given in Table 1. Sixty individuals had their first chickenpox and first stroke/TIA diagnosis in childhood (median age, 3.9 years; interquartile range [IQR], 1.9–6.4 years), and 500 had both chickenpox and first stroke/TIA as an adult (median age, 62.9 years; IQR, 49.2–75.4 years). Just over half the sample in each age category was male. Almost all first episodes in children were strokes, whereas just over half of the adult first episodes were TIAs (Table 1).

**Table 1.** Characteristics of 560 Eligible Patients With Both Chickenpox and a Stroke (or Transient Ischemic Attack) During Follow-up

Characteristic	GPRD		THIN		QResearch		IMS		Total	
	<18 y at Index Date	≥18 y at Index Date	<18 y at Index Date	≥18 y at Index Date	<18 y at Index Date	≥18 y at Index Date	<18 y at Index Date	≥18 y at Index Date	<18 y at Index Date	≥18 y at Index Date
	(n = 17)	(n = 219)	(n = 4)	(n = 79)	(n = 26)	(n = 84)	(n = 13)	(n = 118)	(n = 60)	(N = 500)
Age at index date^a^, y, median (IQR)	4.3 (1.8–6.4)	62.5 (49.0–75.0)	2.1 (1.5–3.0)	64.8 (49.8–76.7)	4.6 (2.2–11.2)	61.3 (51.0–72.0)	3.8 (2.1–6.3)	63.8 (48.4–75.5)	3.9 (1.9–6.4)	62.9 (49.2–75.4)
Male sex, No. (%)	11 (64.7)	127 (58.0)	4 (100)	36 (45.6)	12 (46.2)	41 (48.8)	7 (53.9)	63 (53.4)	34 (56.7)	267 (53.4)
Total observation^b^, y, median (IQR)	6.7 (4.8–12.1)	13.1 (9.6–18.7)	5.8 (4.9–7.3)	15.7 (10.7–18.9)	6.3 (3.4–11.2)	11.8 (8.8–14.9)	9.3 (5.2–12.7)	17.7 (12.9–20.0)	6.6 (4.7–11.7)	14.2 (9.9–18.8)
TIA^c^, No. (%)	1 (5.9)	113 (51.6)	0 (0)	38 (48.1)	6 (23.1)	43 (51.2)	4 (30.8)	65 (55.1)	11 (18.3)	259 (51.8)
Stroke, No. (%)	16 (94.1)	106 (48.4)	4 (100)	41 (51.9)	20 (76.9)	41 (48.8)	9 (69.2)	53 (44.9)	49 (81.7)	241 (48.2)

Abbreviations: GPRD, General Practice Research Database; THIN, The Health Improvement Network; TIA, transient ischemic attack.

^a^ Index date is the date of first stroke or TIA episode in study period.

^b^ Observation is the follow-up during study period.

^c^ Only TIA diagnostic codes were recorded for the illness episode.

**Figure 2.**
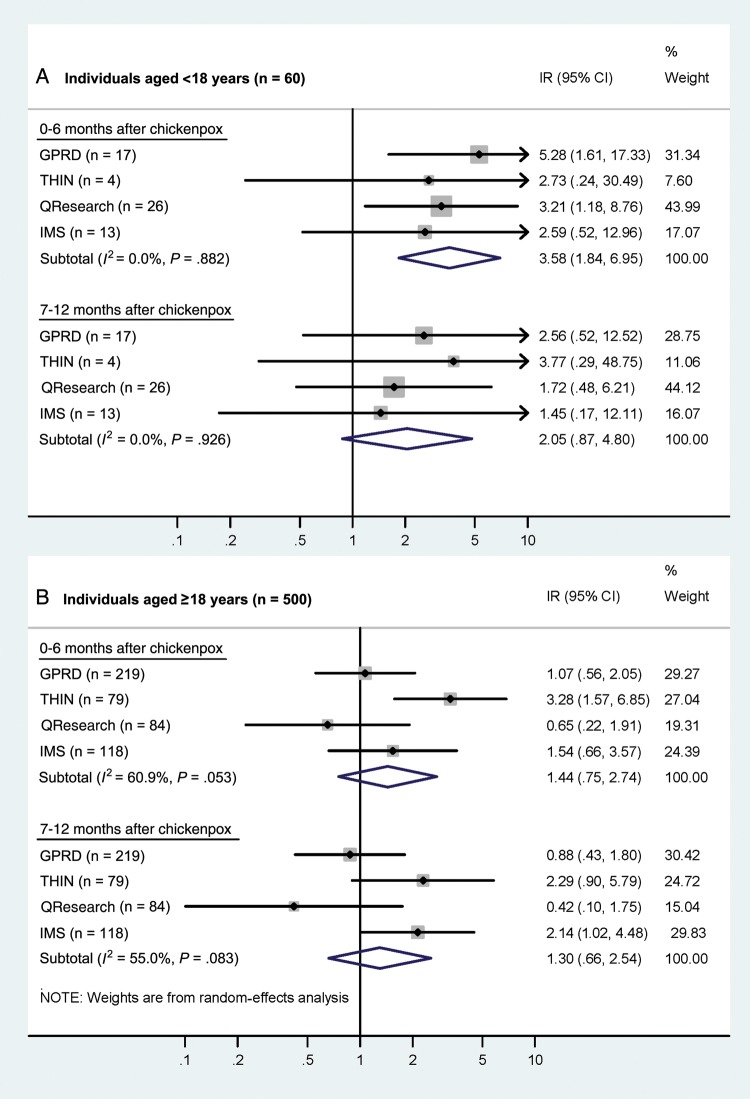
Age-adjusted incidence ratios (IRs) for stroke or transient ischemic attack in periods following chickenpox, in children and adults. For each database, the central diamond and line correspond to the IR and 95% confidence interval, and the area of the gray square reflects the weight of the study. Abbreviations: CI, confidence interval; GPRD, General Practice Research Database; IR, incidence ratio; THIN, The Health Improvement Network.

**Figure 3.**
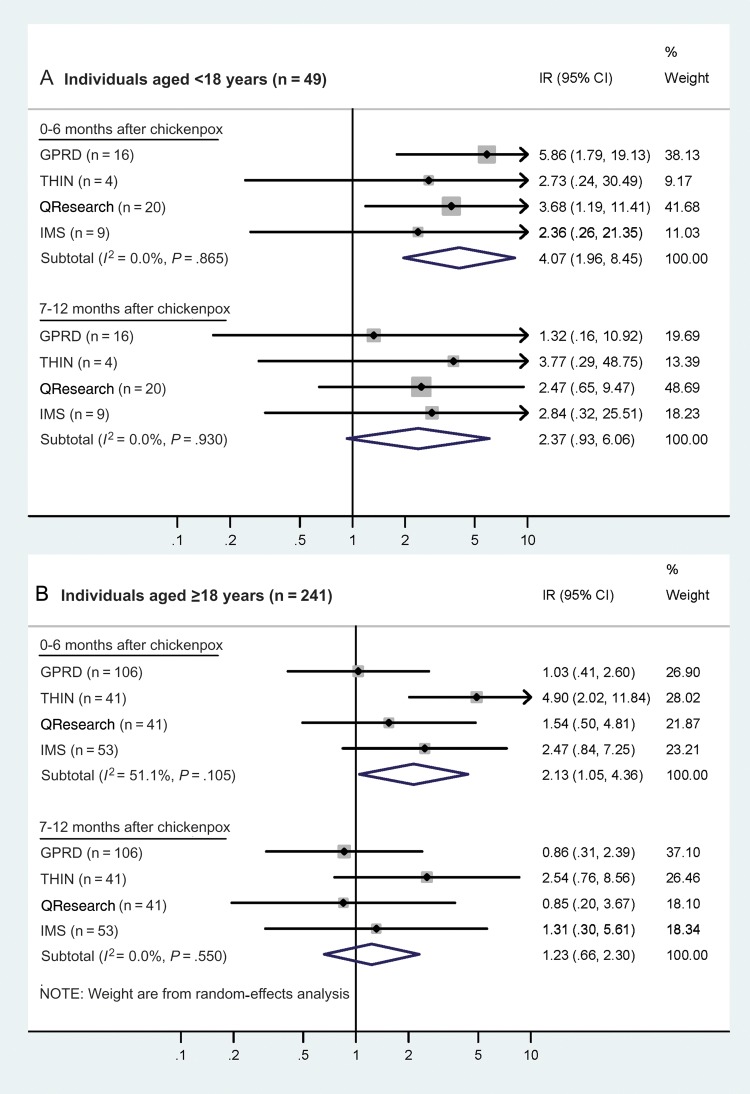
Age-adjusted incidence ratios (IRs) for stroke in periods following chickenpox, in children and adults. For each database, the central diamond and line correspond to the IR and 95% confidence interval, and the area of the gray square reflects the weight of the study. Abbreviations: CI, confidence interval; GPRD, General Practice Research Database; IR, incidence ratio; THIN, The Health Improvement Network.

Results of database-specific analyses and summary effect estimates are given in Figures 2 and 3 (and Supplementary Table 3). Fourteen children experienced a stroke or TIA in the 6 months after chickenpox compared to 39 during baseline periods; an elevated risk was found in all 4 datasets, with no evidence of between-database heterogeneity (*P*_heterogeneity_ = .88; *I*^2^ = 0%) The summary effect estimate (using a fixed-effect model) indicated that children were at a >3-fold increased risk of stroke or TIA in the 6 months following chickenpox (summary IR = 3.58; 95% confidence interval [CI], 1.84–6.95; Figure 2*A*). There was also an indication of elevated stroke/TIA risk 7–12 months after chickenpox, with 7 children experiencing a stroke during this period, although the fixed-effect summary incidence ratio did not reach statistical significance (Figure 2*A*). After combining the data from the 4 datasets and repeating analyses, very similar estimates were obtained (Supplementary Table 3).

In contrast, among adults the incidence ratios for a stroke or TIA in the year after chickenpox were less marked (Figure 2*B*) and varied between datasets (0–6 months: *I*^2^ = 61%; 7–12 months: *I*^2^ = 55%). The results of random-effects meta-analyses provided nonsignificant 44% and 30% increased risks for the 2 post–chickenpox risk periods, with 29 and 23 adults experiencing a stroke 0–6 and 7–12 months after chickenpox, respectively, compared to 448 during baseline.

Effects of chickenpox were more pronounced after restricting analyses to individuals whose first cerebrovascular event was a stroke (Figure 3). Among the 49 children with a stroke, the summary IR in the 0–6 months after chickenpox rose to 4.07 (95% CI, 1.96–8.45; *I*^2^ = 0%), and among the 241 adults the (random-effects) summary IR also rose and reached statistical significance, although moderate between-database heterogeneity remained (IR = 2.13; 95% CI, 1.05–4.36; *I*^2^ = 51%). The summary IRs for strokes in the 7–12 months after chickenpox were not appreciably different from the estimates for strokes/TIAs (Figure 3). Analyses restricted to TIAs were not performed for children as so few had a documented TIA; results from the combined adult data showed little evidence of an association between chickenpox and TIA (0–6 months: IR = 0.85; 95% CI, .43–1.68; Supplementary Table 4).

Repeating analyses after excluding the 1 child and 15 adults whose study period ended <1 month after stroke diagnosis had little effect on the magnitude of effect estimates. The incidence ratio for stroke among children in the 6 months after chickenpox rose very slightly (fixed-effect summary IR = 4.16; 95% CI, 2.00–8.69) and was very similar to the main estimate for the subsequent 6 months (Supplementary Figure 1*A*). Among adults, between-database heterogeneity remained, and random-effects summary estimates were similar to the main findings (Supplementary Figure 1*B*).

## DISCUSSION

Our study shows that among children, chickenpox is associated with an increased risk of stroke in the subsequent 6 months. This supports the assertion that chickenpox is a risk factor for stroke, which until now has been based largely on case reports. We found a less marked nonsignificant increased risk of stroke 7–12 months after chickenpox, further suggesting a reactive process that resolves over time. Among adults, there was less convincing evidence of an increased risk of stroke following chickenpox.

To our knowledge, this is the first robust evidence that chickenpox increases the risk of stroke. One previous case-control study of 11 children with AIS found that 7 (64%) had experienced chickenpox in the previous 9 months compared with 9% of 44 controls, giving a crude odds ratio of 17.5 (95% CI, 2.8–126.1) [18]. The findings were limited by the small sample size and no adjustment for confounding. A second study of 70 childhood AIS cases, with no formal comparison group, reported that 31% had chickenpox in the previous year, 3 times the estimated national population rate [19]. In our study, within-person comparisons meant that confounding was largely addressed. Confounding could have occurred only if individuals had risk factors for stroke that changed over time, if these factors were also associated with the timing of chickenpox, and if these time-dependent effects existed for a high proportion of study participants. Our use of exposed cases, whose chickenpox was diagnosed by a general practitioner, meant that the study did not depend on patient recall and minimized misclassification of chickenpox status. We provide a more precise estimate of increased risk due to our larger number of stroke cases and statistically powerful design. Also, our findings should be highly generalizable, as almost the entire UK population is registered with a general practice.

Very few children presented with a TIA. The differential diagnosis of childhood transient focal neurological deficits is wide, reducing confidence in these events having a definite vascular basis. Diagnosis of TIA may also be imprecise in adults, which could partly explain the lack of a demonstrable association between chickenpox and adult TIA. A further possible explanation relates to the underlying assumption of self-controlled case series methods that clinical events are independent within individuals. As this may not be true for strokes/TIAs, because having 1 stroke/TIA increases the likelihood of another one, we restricted analyses to individuals' first stroke/TIA. This is a reasonable strategy when the event is relatively uncommon during the observation period and has been widely used in previous self-controlled case series stroke studies [2, 15, 20–22]. However, inclusion of TIAs as an additional outcome could have made cerebrovascular events less uncommon, particularly among adults.

Even after restricting analyses among adults to those whose first event was a stroke, there was unexplained between-database heterogeneity in effect estimates. The summary estimate for adults thus needs to be treated with caution, and merits further study. The observed differences in the effect of chickenpox in adults and children may be partly due to mechanistic differences, as childhood AIS is nonatheromatous and focal vasculopathy, probably inflammatory in origin, is likely to play a greater role.

Limitations of our study need consideration. Self-controlled case series analyses only produce estimates of relative incidence, so we could not quantify the absolute risk of stroke following chickenpox. Despite combining data from 4 large general practice databases, our overall sample size was modest, preventing finer stratification of the initial risk period to see if there was a greater elevation of risk within the first few weeks following chickenpox, as has been shown for other infections and stroke [2]. Chickenpox has a highly characteristic clinical presentation and is readily diagnosed by general practitioners. Any misdiagnosis is thus likely to have been at a low level, producing only a small bias toward no effect. Many of the stroke episodes we identified were categorized as “nonspecific,” and some could have been unrecognized hemorrhagic strokes. VZV infection could also increase the risk of cerebral aneurysm and hemorrhage, but this is probably uncommon and inclusion of hemorrhagic strokes may therefore have lessened the association with recent chickenpox [7]. Previous large validation studies in GPRD and THIN have demonstrated high validity of a stroke diagnosis in these data [23, 24]. A small number of events labeled as stroke could potentially have been varicella meningitis or encephalitis. However, we think this is unlikely in children, among whom stroke is an uncommon diagnosis that is not made readily. We did exclude the 1 case who had a varicella meningitis code after a stroke, although this meningitis could have been secondary to cerebral arteriopathy [7]. Our sensitivity analyses also suggested that premature death was unlikely to have affected our study findings. Given our inclusion criteria, our results may not apply to those who have experienced a previous stroke, or to risk of hemorrhagic stroke.

In conclusion, our study provides new evidence that children who experience chickenpox are at increased risk of AIS over the subsequent months and that stroke is a rare complication of childhood chickenpox. The 4-fold increased risk we identified will represent only a small absolute stroke risk, given the low baseline incidence of pediatric stroke. Nevertheless, AIS is increasingly recognized among children and is associated with considerable morbidity and mortality, with up to three-quarters of survivors experiencing neurological and/or cognitive sequelae [25, 26]. Our findings suggest that the renewed attention on the mechanisms by which VZV and other infections cause vascular injury is warranted and could identify strategies to prevent strokes [27].

## Supplementary Data

Supplementary materials are available at *Clinical Infectious Diseases* online (http://cid.oxfordjournals.org/). Supplementary materials consist of data provided by the author that are published to benefit the reader. The posted materials are not copyedited. The contents of all supplementary data are the sole responsibility of the authors. Questions or messages regarding errors should be addressed to the author.

Supplementary Data

## References

[1] Emsley HC, Hopkins SJ (2008). Acute ischaemic stroke and infection: recent and emerging concepts. Lancet Neurol.

[2] Smeeth L, Thomas SL, Hall AJ, Hubbard R, Farrington P, Vallance P (2004). Risk of myocardial infarction and stroke after acute infection or vaccination. N Engl J Med.

[3] Hingorani AD, Cross J, Kharbanda RK (2000). Acute systemic inflammation impairs endothelium-dependent dilatation in humans. Circulation.

[4] Charakida M, Donald AE, Terese M (2005). Endothelial dysfunction in childhood infection. Circulation.

[5] Hills NK, Johnston SC, Sidney S, Zielinski BA, Fullerton HJ (2012). Recent trauma and acute infection as risk factors for childhood arterial ischaemic stroke. Ann Neurol.

[6] Amlie-Lefond C, Bernard TJ, Sebire G (2009). Predictors of cerebral arteriopathy in children with arterial ischemic stroke: results of the International Pediatric Stroke Study. Circulation.

[7] Gilden D, Cohrs RJ, Mahalingam R, Nagel MA (2009). Varicella zoster virus vasculopathies: diverse clinical manifestations, laboratory features, pathogenesis, and treatment. Lancet Neurol.

[8] Miravet E, Danchaivijitr N, Basu H, Saunders DE, Ganesan V (2007). Clinical and radiological features of childhood cerebral infarction following varicella zoster virus infection. Dev Med Child Neurol.

[9] Ciccone S, Faggioli R, Calzolari F, Sartori S, Calderone M, Borgna-Pignatti C (2010). Stroke after varicella-zoster infection: report of a case and review of the literature. Pediatr Infect Dis J.

[10] Ganesan V, Prengler M, McShane MA, Wade AM, Kirkham FJ (2003). Investigation of risk factors in children with arterial ischaemic stroke. Ann Neurol.

[11] Gibbs MA, Fisher M (1986). Cerebral infarction in an adult with disseminated varicella. Bull Clin Neurosci.

[12] Leopold NA (1993). Chickenpox stroke in an adult. Neurology.

[13] Hosseinipour MC, Smith NH, Simpson EP, Greenberg SB, Armstrong RM, White AC (1998). Middle cerebral artery vasculitis and stroke after varicella in a young adult. South Med J.

[14] Kaphan E, Witjas T, Feuillet L (2005). Multiple strokes after chickenpox primo-infection in an adult [in French]. Rev Neurol (Paris).

[15] Whitaker HJ, Farrington CP, Spiessens B, Musonda P (2006). Tutorial in biostatistics: the self-controlled case series method. Stat Med.

[16] Lewis JD, Bilker WB, Weinstein RB, Strom BL (2005). The relationship between time since registration and measured incidence rates in the General Practice Research Database. Pharmacoepidemiol Drug Saf.

[17] Higgins JP, Thompson SG, Deeks JJ, Altman DG (2003). Measuring inconsistency in meta-analyses. BMJ.

[18] Sebire G, Meyer L, Chabrier S (1999). Varicella as a risk factor for cerebral infarction in childhood: a case-control study. Ann Neurol.

[19] Askalan R, Laughlin S, Mayank S (2001). Chickenpox and stroke in childhood: a study of frequency and causation. Stroke.

[20] Farrington CP, Hocine MN (2010). Within-individual dependence in self-controlled case series models for recurrent events. J R Stat Soc C-Appl.

[21] Douglas IJ, Smeeth L (2008). Exposure to antipsychotics and risk of stroke: self controlled case series study. BMJ.

[22] Minassian C, D'Aiuto F, Hingorani AD, Smeeth L (2010). Invasive dental treatment and risk for vascular events: a self-controlled case series. Ann Intern Med.

[23] Herrett E, Thomas SL, Schoonen WM, Smeeth L, Hall AJ (2010). Validation and validity of diagnoses in the General Practice Research Database: a systematic review. Br J Clin Pharmacol.

[24] Ruigomez A, Martin-Merino E, Rodriguez LA (2010). Validation of ischaemic cerebrovascular diagnoses in the health improvement network (THIN). Pharmacoepidemiol Drug Saf.

[25] Heron M (2012). Deaths: leading causes for 2009.

[26] Goldenberg NA, Bernard TJ, Fullerton HJ, Gordon A, deVeber G (2009). Antithrombotic treatments, outcomes, and prognostic factors in acute childhood-onset arterial ischaemic stroke: a multicentre, observational, cohort study. Lancet Neurol.

[27] Fullerton HJ, Elkind MS, Barkovich AJ (2011). The Vascular effects of Infection in Pediatric Stroke (VIPS) study. J Child Neurol.

