# Supplementary material for: Chickenpox and Risk of Stroke: A Self-controlled Case Series Analysis
Source: Clin Infect Dis. 2013 Oct 2;58(1):61–8. doi: 10.1093/cid/cit659 (PMC3864501; doi:10.1093/cid/cit659)
Supplement: Supplementary Data [file supp_58_1_61__index.html]

Chickenpox and risk of stroke: a self-controlled case series analysis — Chickenpox and Risk of Stroke: A Self-controlled Case Series Analysis — Chickenpox and Risk of Stroke: A Self-controlled Case Series Analysis — Supplementary Data 

# Chickenpox and Risk of Stroke: A Self-controlled Case Series Analysis

## Supplementary Data

Supplementary Data

**Files in this Data Supplement:**

- Supplementary Figure 1 - pdf file
- Supplementary Table 1 - doc file
