# Supplementary material for: Chickenpox and Risk of Stroke: A Self-controlled Case Series Analysis
Source: Clin Infect Dis. 2013 Oct 2;58(1):61–8. doi: 10.1093/cid/cit659 (PMC3864501; doi:10.1093/cid/cit659)

Supplementary Figure 1: Age-adjusted incidence ratios (IR) for non-fatal stroke in periods following chickenpox, in children and adults. For each database, the central diamond and line correspond to the IR and 95% CI, and the area of the grey square reflects the weight of the study.

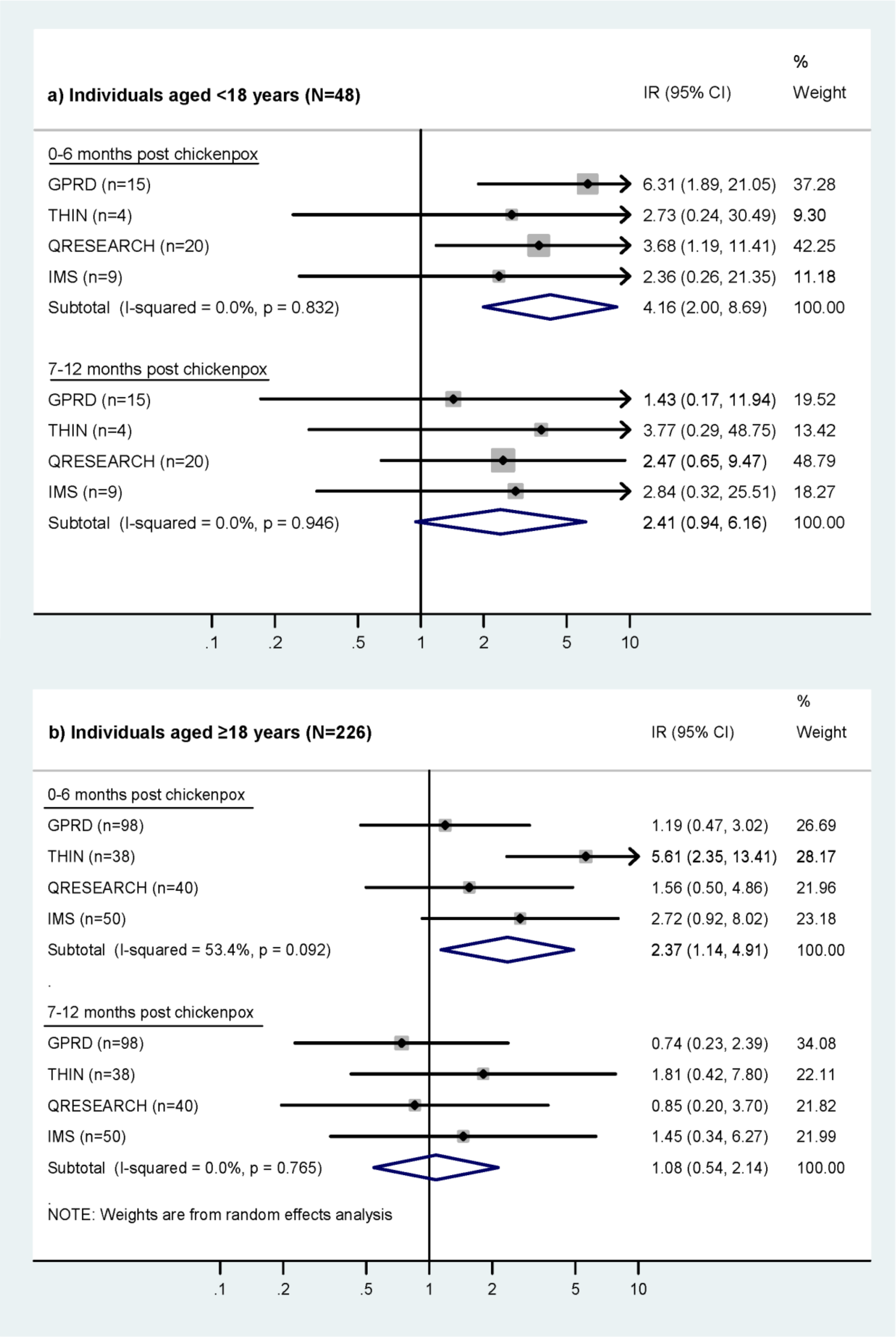

Supplement: Supplementary Data [file supp_cit659_cit659supp_fig1.pdf]
