# Supplementary material for: Chickenpox and Risk of Stroke: A Self-controlled Case Series Analysis
Source: Clin Infect Dis. 2013 Oct 2;58(1):61–8. doi: 10.1093/cid/cit659 (PMC3864501; doi:10.1093/cid/cit659)
Supplement: Supplementary Data [file supp_cit659_cit659supp_table1.doc]

e-Table 1: Read codes for chickenpox

**b) Individuals aged ≥18 years (N=500)**

**a) Individuals aged <18 years (N=60)**

**a) Individuals aged <18 years (N=60)**

| **Read code** | **Description** |
| --- | --- |
| **A52..00** | Chickenpox - varicella |
| **A52..11** | Chickenpox |
| **A520.00** | Postvaricella encephalitis |
| **A521.00** | Varicella pneumonitis |
| **A52x.00** | Varicella with other specified complications |
| **A52y.00** | Varicella with unspecified complications NOS |
| **A52z.00** | Varicella with no complication NOS |
| **AyuA300** | [X]Varicella without complications |
| **F011700** | Varicella meningitis |
| **F035000** | Encephalitis following chickenpox |
| **F035011** | Encephalitis due to varicella |
| **F037000** | Varicella transverse myelitis |
| **H24y700** | Pneumonia with varicella |

**e-Table 2: Read codes for arterial ischemic stroke, stroke of unspecified type and transient ischemic attack (TIA)a**

| **Read code** | **Description** | **tia** |
| --- | --- | --- |
| **Fyu5600** | [X]Other lacunar syndromes | 0 |
| **G63..00** | Precerebral arterial occlusion | 0 |
| **G63..11** | Infarction - precerebral | 0 |
| **G630.00** | Basilar artery occlusion | 0 |
| **G631.00** | Carotid artery occlusion | 0 |
| **G631.12** | Thrombosis, carotid artery | 0 |
| **G632.00** | Vertebral artery occlusion | 0 |
| **G633.00** | Multiple and bilateral precerebral arterial occlusion | 0 |
| **G63y.00** | Other precerebral artery occlusion | 0 |
| **G63y000** | Cerebral infarct due to thrombosis of precerebral arteries | 0 |
| **G63y100** | Cerebral infarction due to embolism of precerebral arteries | 0 |
| **G63z.00** | Precerebral artery occlusion NOS | 0 |
| **G64..00** | Cerebral arterial occlusion | 0 |
| **G64..11** | CVA - cerebral artery occlusion | 0 |
| **G64..12** | Infarction - cerebral | 0 |
| **G64..13** | Stroke due to cerebral arterial occlusion | 0 |
| **G640.00** | Cerebral thrombosis | 0 |
| **G640000** | Cerebral infarction due to thrombosis of cerebral arteries | 0 |
| **G641.00** | Cerebral embolism | 0 |
| **G641.11** | Cerebral embolus | 0 |
| **G641000** | Cerebral infarction due to embolism of cerebral arteries | 0 |
| **G64z.00** | Cerebral infarction NOS | 0 |
| **G64z.11** | Brainstem infarction NOS | 0 |
| **G64z.12** | Cerebellar infarction | 0 |
| **G64z000** | Brainstem infarction | 0 |
| **G64z100** | Wallenberg syndrome | 0 |
| **G64z111** | Lateral medullary syndrome | 0 |
| **G64z200** | Left sided cerebral infarction | 0 |
| **G64z300** | Right sided cerebral infarction | 0 |
| **G64z400** | Infarction of basal ganglia | 0 |
| **G650.00** | Basilar artery syndrome | 0 |
| **G651.00** | Vertebral artery syndrome | 0 |
| **G651000** | Vertebro-basilar artery syndrome | 0 |
| **G653.00** | Carotid artery syndrome hemispheric | 0 |
| **G654.00** | Multiple and bilateral precerebral artery syndromes | 0 |
| **G66..00** | Stroke and cerebrovascular accident unspecified | 0 |
| **G66..11** | CVA unspecified | 0 |
| **G66..12** | Stroke unspecified | 0 |
| **G66..13** | CVA - Cerebrovascular accident unspecified | 0 |
| **G660.00** | Middle cerebral artery syndrome | 0 |
| **G661.00** | Anterior cerebral artery syndrome | 0 |
| **G662.00** | Posterior cerebral artery syndrome | 0 |
| **G663.00** | Brain stem stroke syndrome | 0 |
| **G664.00** | Cerebellar stroke syndrome | 0 |
| **G665.00** | Pure motor lacunar syndrome | 0 |
| **G666.00** | Pure sensory lacunar syndrome | 0 |
| **G667.00** | Left sided CVA | 0 |
| **G668.00** | Right sided CVA | 0 |
| **G669.00** | Cerebral palsy, not congenital or infantile, acute | 0 |
| **G671000** | Acute cerebrovascular insufficiency NOS | 0 |
| **G677000** | Occlusion and stenosis of middle cerebral artery | 0 |
| **G677100** | Occlusion and stenosis of anterior cerebral artery | 0 |
| **G677200** | Occlusion and stenosis of posterior cerebral artery | 0 |
| **G677300** | Occlusion and stenosis of cerebellar arteries | 0 |
| **G677400** | Occlusion+stenosis of multiple and bilat cerebral arteries | 0 |
| **G6W..00** | Cereb infarct due unsp occlus/stenos precerebr arteries | 0 |
| **G6X..00** | Cerebrl infarctn due/unspcf occlusn or sten/cerebrl artrs | 0 |
| **Gyu6300** | [X]Cerebrl infarctn due/unspcf occlusn or sten/cerebrl artrs | 0 |
| **Gyu6400** | [X]Other cerebral infarction | 0 |
| **Gyu6500** | [X]Occlusion and stenosis of other precerebral arteries | 0 |
| **Gyu6600** | [X]Occlusion and stenosis of other cerebral arteries | 0 |
| **Gyu6G00** | [X]Cereb infarct due unsp occlus/stenos precerebr arteries | 0 |
| **L440.11** | CVA - cerebrovascular accident in the puerperium | 0 |
| **L440.12** | Stroke in the puerperium | 0 |
| **Fyu5500** | [X]Other transnt cerebral ischemic attacks+related syndroms | 1 |
| **G65..00** | Transient cerebral ischaemia | 1 |
| **G65..12** | Transient ischaemic attack | 1 |
| **G65y.00** | Other transient cerebral ischaemia | 1 |
| **G65z.00** | Transient cerebral ischaemia NOS | 1 |
| **G65z000** | Impending cerebral ischaemia | 1 |
| **G65z100** | Intermittent cerebral ischaemia | 1 |
| **G65zz00** | Transient cerebral ischaemia NOS | 1 |

^a^Excludes codes indicating a history of stroke

**e-Table 3.** Age-adjusted incidence ratios for stroke/TIA in risk periods following chickenpox

| **Outcome**  *risk period post-chickenpox* | Cases <18 yrs | | | Cases *≥*18 yrs | | |
| --- | --- | --- | --- | --- | --- | --- |
|  | In risk  period  (n) | Baseline^a^  (n) | IR (95% CI) | In risk  period  (n) | Baseline^a^  (n) | IR (95% CI) |
| **Stroke or TIA** |  |  |  |  |  |  |
| *0-6 months* |  |  |  |  |  |  |
| GPRD | 5 | 10 | 5.28 (1.61-17.33) | 10 | 201 | 1.07 (0.56-2.05) |
| THIN | 1 | 2 | 2.73 (0.24-30.49) | 9 | 65 | 3.28 (1.57-6.85) |
| QResearch | 6 | 17 | 3.21 (1.18-8.76) | 4 | 78 | 0.65 (0.22-1.91) |
| IMS | 2 | 10 | 2.59 (0.52-12.96) | 6 | 104 | 1.54 (0.66-3.57) |
| Meta-analysis^b^ | 14 | 39 | 3.58 (1.84-6.95) | 29 | 448 | 1.44 (0.75-2.74) |
| Combined analysis^c^ | 14 | 39 | 3.42 (1.78-6.56) | ‡^c^ | ‡^c^ | ‡^c^ |
| *7-12 months* |  |  |  |  |  |  |
| GPRD | 2 | 10 | 2.56 (0.52-12.52) | 8 | 201 | 0.88 (0.43-1.80) |
| THIN | 1 | 2 | 3.77 (0.29-48.75) | 5 | 65 | 2.29 (0.90-5.79) |
| QResearch | 3 | 17 | 1.72 (0.48-6.21) | 2 | 78 | 0.42 (0.10-1.75) |
| IMS | 1 | 10 | 1.45 (0.17-12.11) | 8 | 104 | 2.14 (1.02-4.48) |
| Meta-analysis^b^ | 7 | 39 | 2.05 (0.87-4.80) | 23 | 448 | 1.30 (0.66-2.54) |
| Combined analysis^c^ | 7 | 39 | 1.92 (0.84-4.42) | ‡^c^ | ‡^c^ | ‡^c^ |
| **Stroke** |  |  |  |  |  |  |
| *0-6 months* |  |  |  |  |  |  |
| GPRD | 5 | 10 | 5.86 (1.79-19.13) | 5 | 97 | 1.03 (0.41-2.60) |
| THIN | 1 | 2 | 2.73 (0.24-30.49) | 7 | 31 | 4.90 (2.02-11.84) |
| QResearch | 5 | 12 | 3.68 (1.19-11.41) | 4 | 35 | 1.54 (0.50-4.81) |
| IMS | 1 | 7 | 2.36 (0.26-21.35) | 4 | 47 | 2.47 (0.84-7.25) |
| Meta-analysis^b^ | 12 | 31 | 4.07 (1.96-8.45) | 20 | 210 | 2.13 (1.05-4.36) |
| Combined analysis^c^ | 12 | 31 | 3.89 (1.91-7.92) | ‡^c^ | ‡^c^ | ‡^c^ |
| *7-12 months* |  |  |  |  |  |  |
| GPRD | 1 | 10 | 1.32 (0.16-10.92) | 4 | 97 | 0.86 (0.31-2.39) |
| THIN | 1 | 2 | 3.77 (0.29-48.75) | 3 | 31 | 2.54 (0.76-8.56) |
| QResearch | 3 | 12 | 2.47 (0.65-9.47) | 2 | 35 | 0.85 (0.20-3.67) |
| IMS | 1 | 7 | 2.84 (0.32-25.51) | 2 | 47 | 1.31 (0.30-5.61) |
| Meta-analysis^b^ | 6 | 31 | 2.37 (0.93-6.06) | 11 | 210 | 1.23 (0.66-2.30) |
| Combined analysis^c^ | 6 | 31 | 2.20 (0.89-5.46) | ‡^c^ | ‡^c^ | ‡^c^ |

IR denotes age-adjusted incidence ratio.

^a^ Baseline period is all observation time except for 12 month period starting on day after chickenpox.

^b^Random effects for cases >=18 yrs, fixed effect for cases <18 years.

^c^Combined analyses not done for cases ***≥***18 yrs due to between-database heterogeneity.

**e-Table 4.** Age-adjusted incidence ratios (IR) for TIA in the 0-6 months following chickenpox, in adults (n=259)

| *risk period post-chickenpox* | Cases *≥*18 yrs | | |
| --- | --- | --- | --- |
|  | In risk  period  (n) | Baseline  (n) | IR (95% CI) |
| **TIA** |  |  |  |
| *0-6 months* |  |  |  |
| GPRD | 5 | 104 | 1.06 (0.42-2.69) |
| THIN | 2 | 34 | 1.60 (0.37-6.98) |
| QResearch | 0 | 43 | †^a^ |
| IMS | 2 | 57 | 0.96 (0.23-4.01) |
| Combined analysis^b^ | 9 | 238 | 0.85 (0.43-1.68) |

^a^ Incidence ratio not calculated, as all TIAs occurred during baseline

^b^Meta-analysis was not used to combine data, due to missing incidence ratio for QResearch

**a) Individuals aged <18 years (N=48)**

**b) Individuals aged ≥18 years (N=226)**
